# Source discrimination of mine water based on the random forest method

**DOI:** 10.1038/s41598-022-24037-4

**Published:** 2022-11-15

**Authors:** Zhenwei Yang, Hang Lv, Zhaofeng Xu, Xinyi Wang

**Affiliations:** 1 Institute of Resources and Environment, Henan Polytechnic University, Jiaozuo, 454000 China; 2 Collaborative Innovation Center of Coal Work Safety and Clean High Efficiency Utilization, Jiaozuo, 454000 China; 3 State Key Laboratory of Coking Coal Exploitation and Comprehensive Utilization, China Pingmei Shenma Holding Group Co., LTD, Pingdingshan, 467000 China

**Keywords:** Hydrology, Hydrogeology

## Abstract

Machine learning is one of the widely used techniques to pattern recognition. Use of the machine learning tools is becoming a more accessible approach for predictive model development in preventing engineering disaster. The objective of the research is to for estimation of water source using the machine learning tools. Random forest classification is a popular machine learning method for developing prediction models in many research settings. The type of mine water in the Pingdingshan coalfield is classified into surface water, Quaternary pore water, Carboniferous limestone karst water, Permian sandstone water, and Cambrian limestone karst water. Each type of water is encoded with the number 0–4. On the basis of hydrochemical data processing, a random forests model is designed and trained with the hydrochemical data. With respect to the predictive accuracy and robustness, fourfold cross-validation (CV) is adopted for the model training. The results show that the random forests model presented here provides significant guidance for the discrimination of mine water.

## Introduction

Coal is the most important energy source in China. The mine safety production is associated with the sustainable development and economic stability. The mine hydrogeological conditions are complicated^1^. With the increasing depth of coal mining, the source of mine water inrush becomes increasingly complex. It can lead to serious disasters due to the complicated hydrogeological conditions found in parts of China, which are uncommon elsewhere in the world. Therefore, rapid and accurate discrimination of the source of water inrush is very important and necessary for both resuming production and rescuing miners^2^.

The hydrochemical composition maintains an equilibrium, even though a series of chemical and physical reactions such as redox, precipitation and dissolution occur constantly between rock and groundwater^3^. Consequently, the chemical characteristics of groundwater in different aquifers are distinct, and the same aquifer is consistent, which is the basis of the source discrimination of mine water derived from hydrochemical characteristics. Many mathematical models of mine water source discrimination have been well established over the past several decades^4,5^. For example, cluster analysis, distance discrimination, grey analysis, bayes, fuzzy evaluation, and so on. Based on mathematical methods, hydrochemistry is widely used to identify mine water sources. With the development of machine learning, more and more research on source discrimination of mine water has been conducted by artificial intelligence, such as BP neural network, deep learning and support vector machines (SVM)^6,7^.

It is a beneficial attempt to apply mathematical models and artificial intelligence to source discrimination of mine water. There are some limitations to these methods. (1) Most of the mathematical models focus on two or more values, and the data distribution ranges greatly, which is difficult to process correctly. (2) Generally, the number of water samples is several dozen or even hundreds^8,9^. The data is abundant for model training by BP neural network and SVM, but it is not easy to be operated by these methods. For deep learning, thousands of data samples are needed for the model training. Obviously, it is far from enough for deep learning^10,11^. As a fast, flexible, and representative method for mining high dimensional data, random forest is a commonly used machine learning algorithm trademarked by Leo Breiman and Adele Cutler, which performs well even in the presence of a large number of features and a small number of observations^12,13^.

The main contribution of this research is (1) to introduce random forests into source discrimination of mine water to build a discriminant model and (2) to train the model parameters and apply it to water source discrimination in the Pingdingshan coal field. The objective of the study is to develop new ideas for the discrimination of water inrush sources. The organization of the paper is as follows. “Geological conditions and hydrogeological data” presents the geological and hydrogeological conditions of the study area. The source discrimination of mine water problems in the framework of the random forest is introduced in detail in “Methodology”. The implementation procedure is introduced in “Implementation procedure”. The results and discussion for the source discrimination of mine water are demonstrated in “Results and discussion”. This paper closes with some conclusions and final remarks.

## Geological conditions and hydrogeological data

### Outline of the coalfield

**Figure 1:**
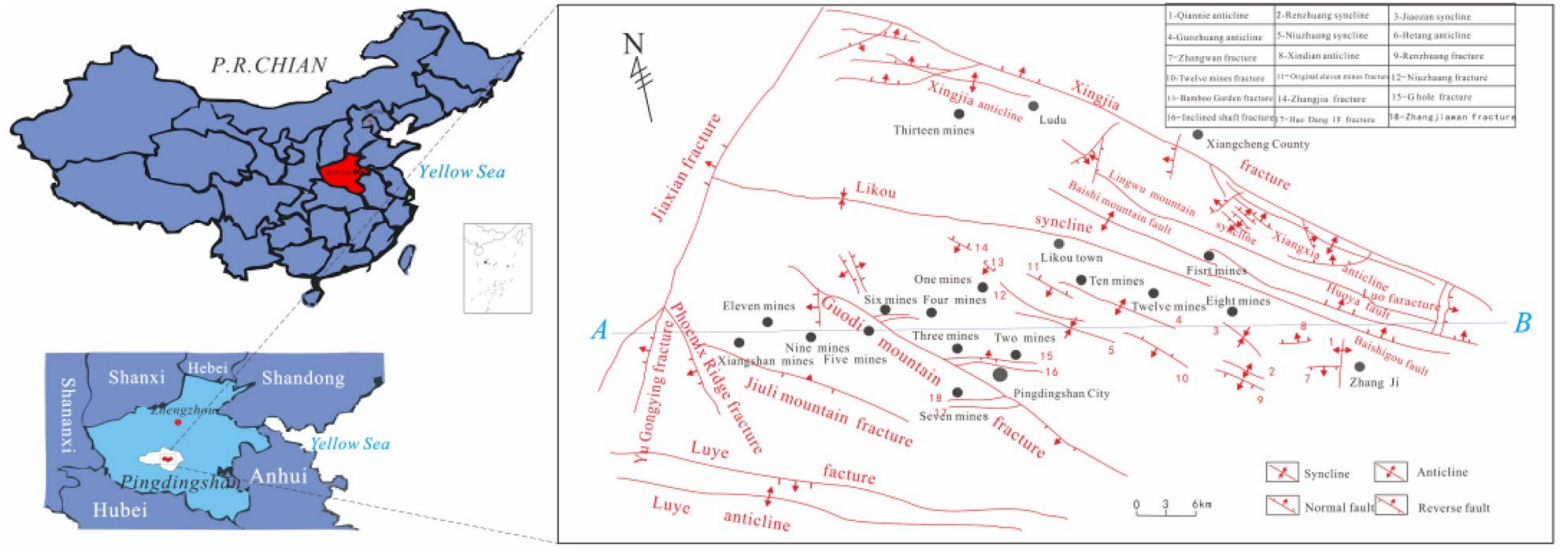
General map of the study area (the figure was drawn by MapGIS 6.7, URL link: https://www.mapgis.com/index.php?a=shows&catid=97&id=29).

**Figure 2:**
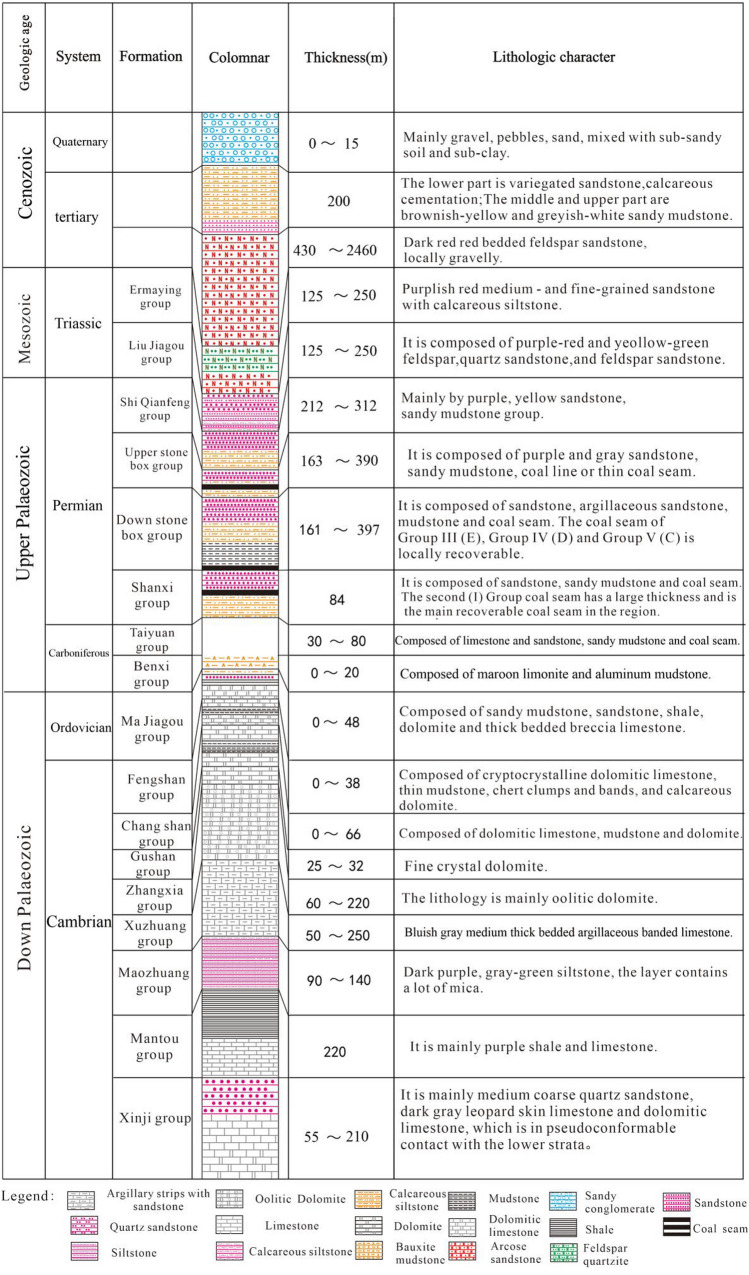
Comprehensive histogram of strata in the Pingdingshan coalfield.

The Pingdingshan coalfield is located in the central and western parts of Henan Province, northern China (Fig. 1), which is the third largest coal producer in China. The length is about 40 km long E–W and 20 km wide N–S. There are 17 coal mines occupied a total area of about 400 km^2^ at the coalfield. The studied area can be divided into eastern and western areas by the Guodishan fault. It is a large syncline with symmetrically gently dipping limbs^14^. The coal-bearing sediments are mostly Permian in age, comprised of sandstone, siltstone and carbonaceous shale. They are overlaid by Neogene, Paleogene and Quaternary deposits (Fig. 2).

### Hydrogeological conditions

The study area is situated in a transitional zone from a warm temperate zone to a subtropical zone, with a long-term average precipitation of 747.4 mm/year, mainly concentrated from July to September. With a surface elevation varying from 900 to 1040 m, the topography is low in the southeast and high in the northwest. Influenced by the topographical features, the rivers, such as the Shahe, Ruhe, Zhanhe and Baiguishan Reservoir, are mainly distributed in the south and north of the mining area. There are some other seasonal rivers and man-made ditches, such as Zhanhe, Beigan Canal and Xigan Canal. The riverbed inserts into Cambrian limestone or Neogene marl, which has a certain replenishment effect on the groundwater of limestone in the No.7 mine in the southwest of the Pingdingshan coalfield^15,16^.

There are four main water filled aquifers in the study area. From the upper to the bottom, mainly include:(1) The Quaternary sand gravel pore aquifer, which covers the coal strata, contacts the minable seam on the outcrop. The osmotic coefficient is 0.000626 m/day. (2) Dyas sandstone aquifer, composed by medium sized and large sandstones, has poor water yield and poor supplementation conditions. (3) The Taiyuan formation of the Carboniferous system. There are seven layers of limestone in the formation. Most of them are dominated by corrosion fissures. The supplementation condition is poor. The water inflow per unit is 0.00018–0.3569 L/s m, and the permeability coefficient is 0.0076–3.047 m/day. (4) The middle and upper Canmbrian limestone aquifer, which is the indirect water-filled aquifer of the upper coalbed. The thick dolomite limestone of the upper Gushan Formation and the thick oolith limestone in the upper Zhangxia Formation are predominant in this layer. The osmotic coefficient of 1.092–7.47 m/day and the unit-specific capacity is 2.27–26.62 l/s m^17^.

### Dataset

**Figure 3:**
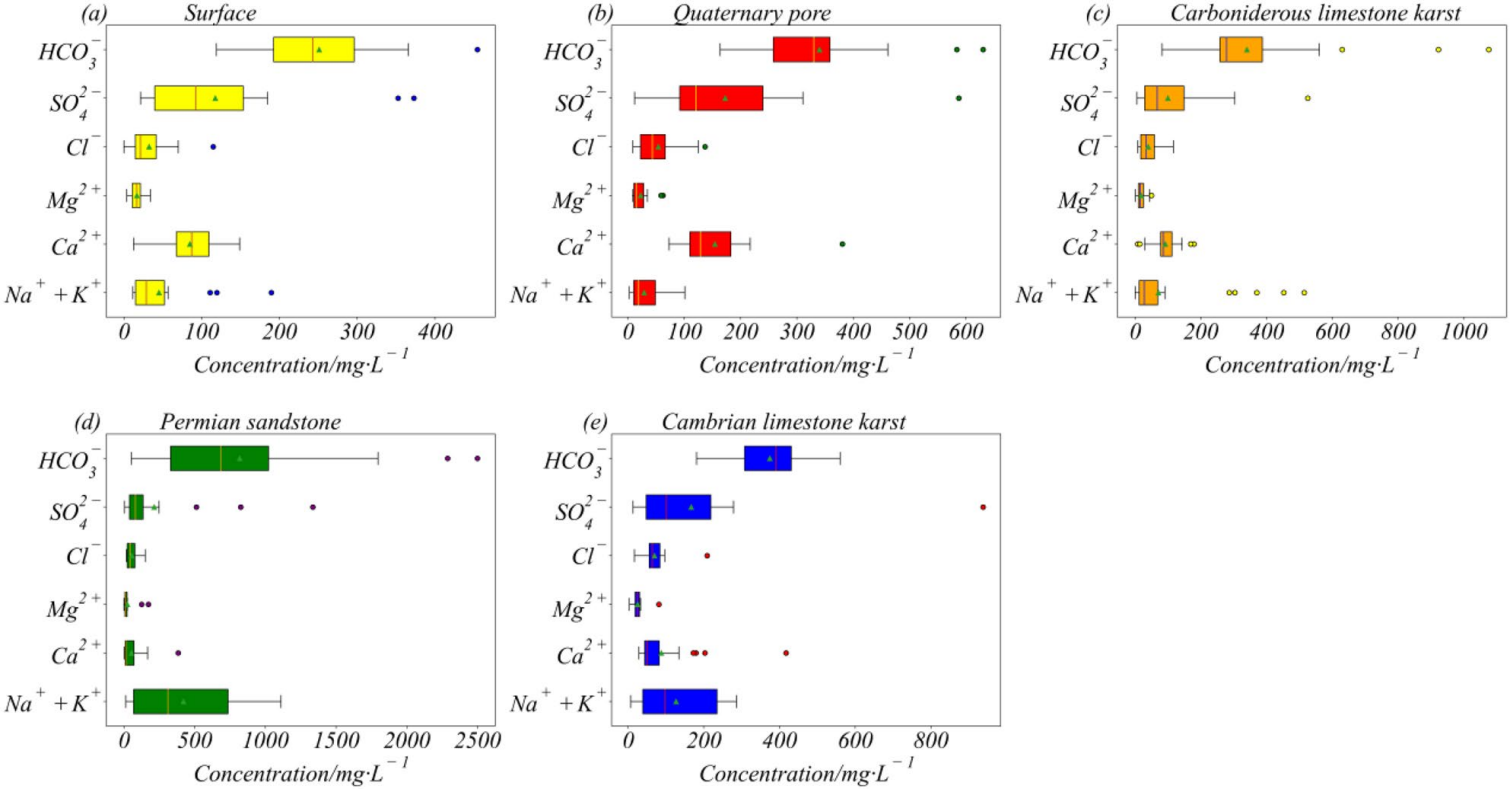
Boxplots of major hydrochemical parameters for different aquifers.

In the study, one hundred and forty-nine mine water samples were collected. All samples were sent to the laboratory as soon as possible for further analysis. The box plots in Fig. 3 shows the characteristics of the original data distribution, which compares multiple parameters for the same aquifer. As a whole, the range of HCO- 3 content changes more greatly than other ion compositions in all the aquifers. The Mg^2+^ concentration is significantly higher than other ions.

Data standardization is about ensuring that data is internally consistent, that is, each data type has the same content and format. Standardized values are useful for tracking data that isn’t easy to compare otherwise. The raw data are normalized individually according to Eq. (1).

1 $$Z_{ij} = \left( {x_{ij} - {\text{mean}}\left( {x_{j} } \right)} \right)/{\text{std}}\left( {x_{j} } \right)$$

where the subscript *i* means the row of the data matrix, the subscript *j* means the column of the data matrix, *Z*_*ij*_ represents the data after standardization, *x*_*ij*_ represents the source data, and the symbol std represents the standard deviation of related data.

**Figure 4:**
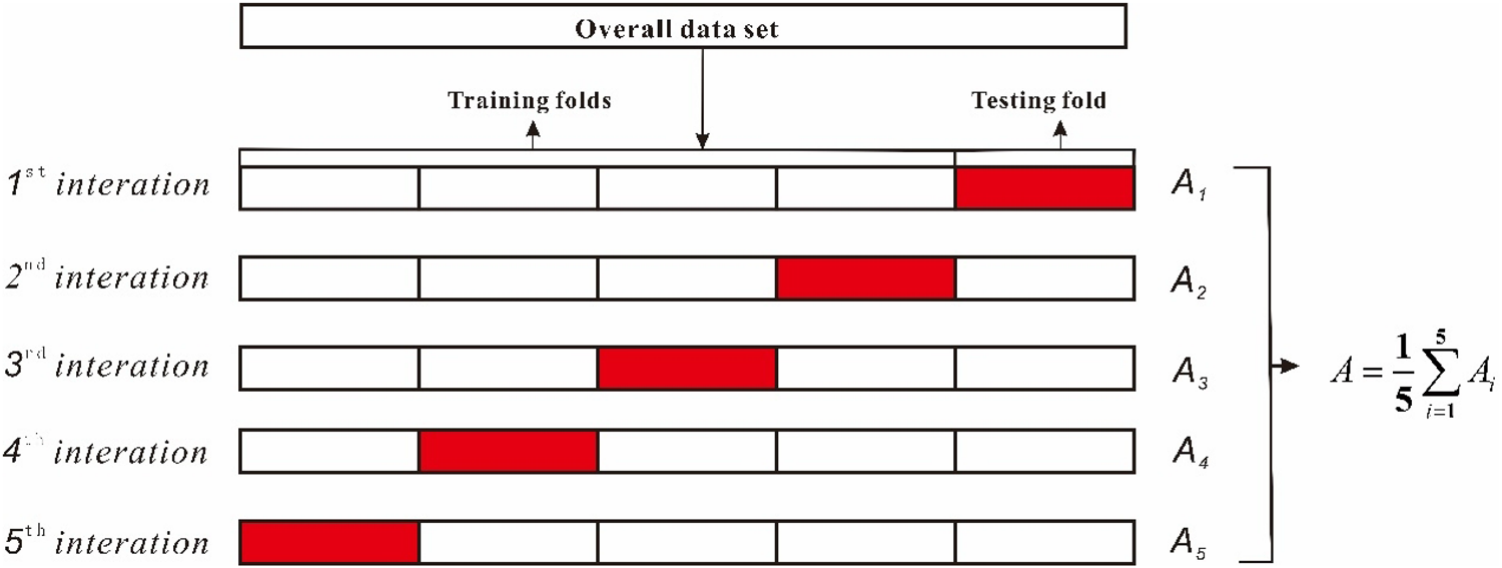
The schematic diagram of Random forests model.

In theory, the dataset could be split into three subsets: training set, validation set, and testing set. The training set is utilized to training the model; the validation set is used to estimate prediction error for model selection; and the testing set is adopted to assess the generalization error of the finalized model. If there is enough data at hand, the best practice is to randomly split. Because our data is generally scarce, the inability to truly reflect the generalization performance of the model is common. In order to avoid any bias in data selection, k-fold Cross-Validation (CV) was employed in the paper during the process of hyper-parameters tuning and model assessment^5^. In k-fold CV, original samples S are randomly split into k mutually exclusive subsets of similar size, i.e. S = S_1_ ∪ S_2_ ∪ … ∪ S_k_, S_*i*_ ∩ S_*j*_ = Ø{*i* ≠ *j*}. Each subset S_*i*_ maintains the consistency of the data distribution as much as possible, that is, from hierarchical sampling of S. Then, each time the union of k subsets is used as the training set, and the remaining subset is utilized as the testing set; therefore, the k group training and testing dataset can be obtained, and k training and testing cross-validation can be performed. There is no definite strict rule for determining the value of *k*. A value of *k* = 5 is very common in the field of random forest. In the aspect, the number of k is set to 5 and associated with the trade-off between the bias and the computation time. Thus, the manuscript adopts the method of fivefold cross-validation to train the model (Fig. 4).

## Methodology

### Random forests (RF)

Random forests, designed for statistical learning, is one of the most famous machine learning approaches. The randomness is reflected in two aspects, one is random selection of features, the other is random sampling, so that each tree in the forest has both similarities and differences. As a supervised learning methodology, it employs a number of decision trees and generally uses the bootstrap resampling method to extract multiple samples from original samples. Each tree in the classifications takes input from samples in the original dataset, and all of features are selected randomly, which are used in growing the tree at every node^18,19^. With similar distribution in the random forest, each tree is dependent on random vectors sampled independently. Trees in the forest will not be pruned until the end of the exercise when the prediction is reached decisively. Combining the predictions of multiple decision trees produces an average for the final forecast results^20^.

**Figure 5:**
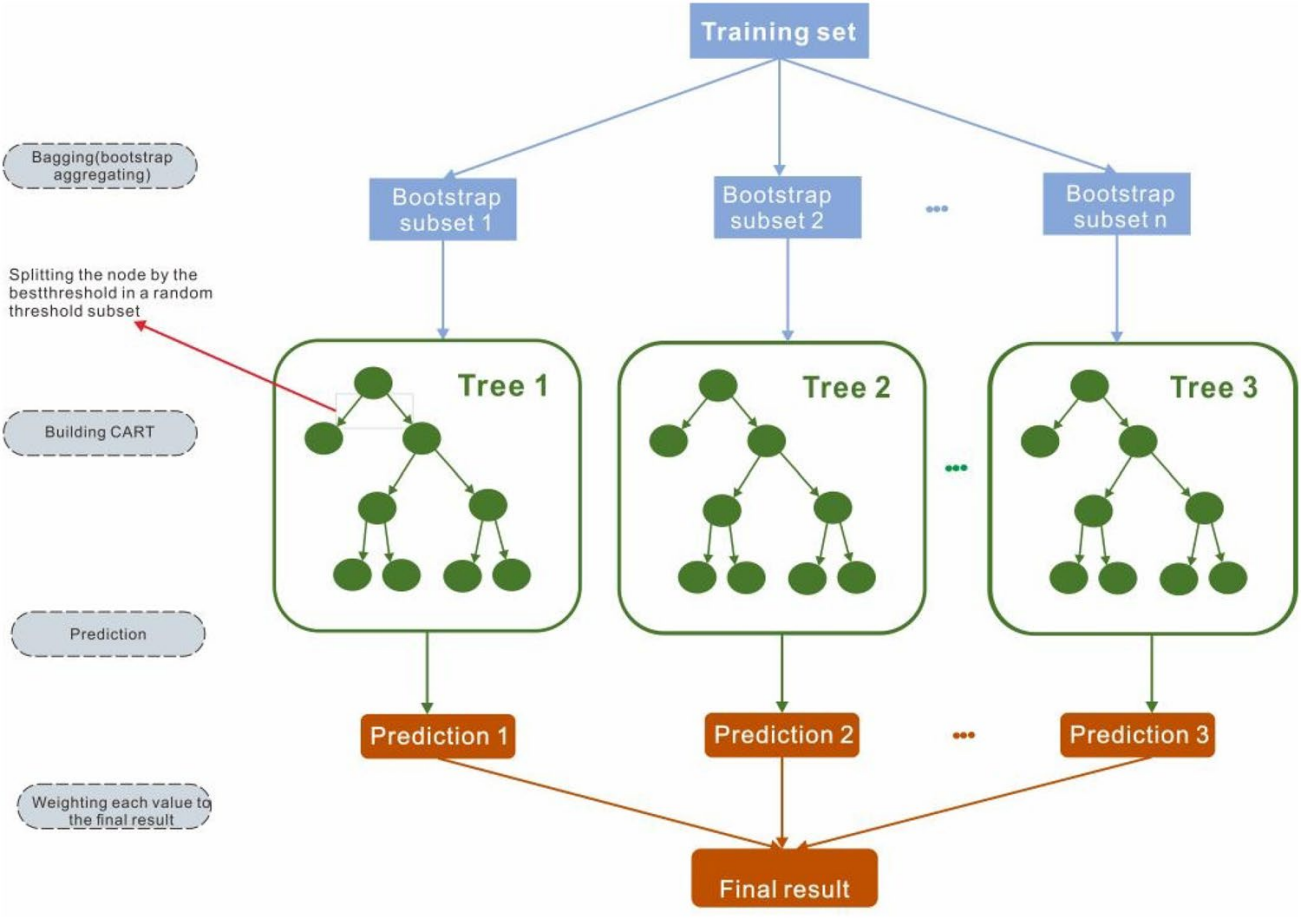
The algorithmic diagram of random forests model.

The schematic of random forest model can be seen in Fig. 5. The training set should be constructed at the beginning. Each tree training in the sample uses random subsets from the initial training samples. Then, the subsets are used as the input to the classification and regression tree (CART). At each node of the random tree, *m* features are selected at random out of the initial features, and the optimal split is chosen from the randomly selected features of the unpruned tree nodes. Each tree grows without limits and should not be pruned whatsoever. Finally, predictions and results are weighted over trees by taking the majority vote over all trees^8^.

### Performance measures

The RMSE (root mean square error) is employed to analyze and assess the predictive results of the machine learning models (Eq. 2). The value closer to 0 indicates that the error in prediction is less^22^.

2 $${\text{RMSE}} = \sqrt {\frac{1}{n}\mathop \sum \limits_{i = 1}^{n} \left( {y_{i} - \hat{y}_{i} } \right)^{2} }$$

### Variable importance measurement (VIM)

In order to quantitatively calculate the effect of every factor on the source discrimination of mine water, the mean decrease impurity importance (MDI) method is used to measure the variable importance, which is constructed in the following way. In the study of forest, the importance of a variable Vi could be evaluated by adding up the weighted impurity decreases q(t)∆ j(st,t) for the whole trees *φ*_*n*_(form = 1,…,*N*) in the forest:

3 $$Im(V_{i} ) = \frac{1}{N}\sum\limits_{n = 1}^{N} {\sum\limits_{{k = \varphi_{n} }} {1(i_{k} = i)[q(k)\Delta j(s_{k} ,k)]} }$$

where $q(k) = \frac{{m_{k} }}{m}$ is the proportion of samples, *i*_*k*_ is the identifier of the variable used for splitting node *k*, and ∆*j* denotes the decrease impurity, which is the value of RMSE for the prediction^23^.

## Implementation procedure

**Figure 6:**
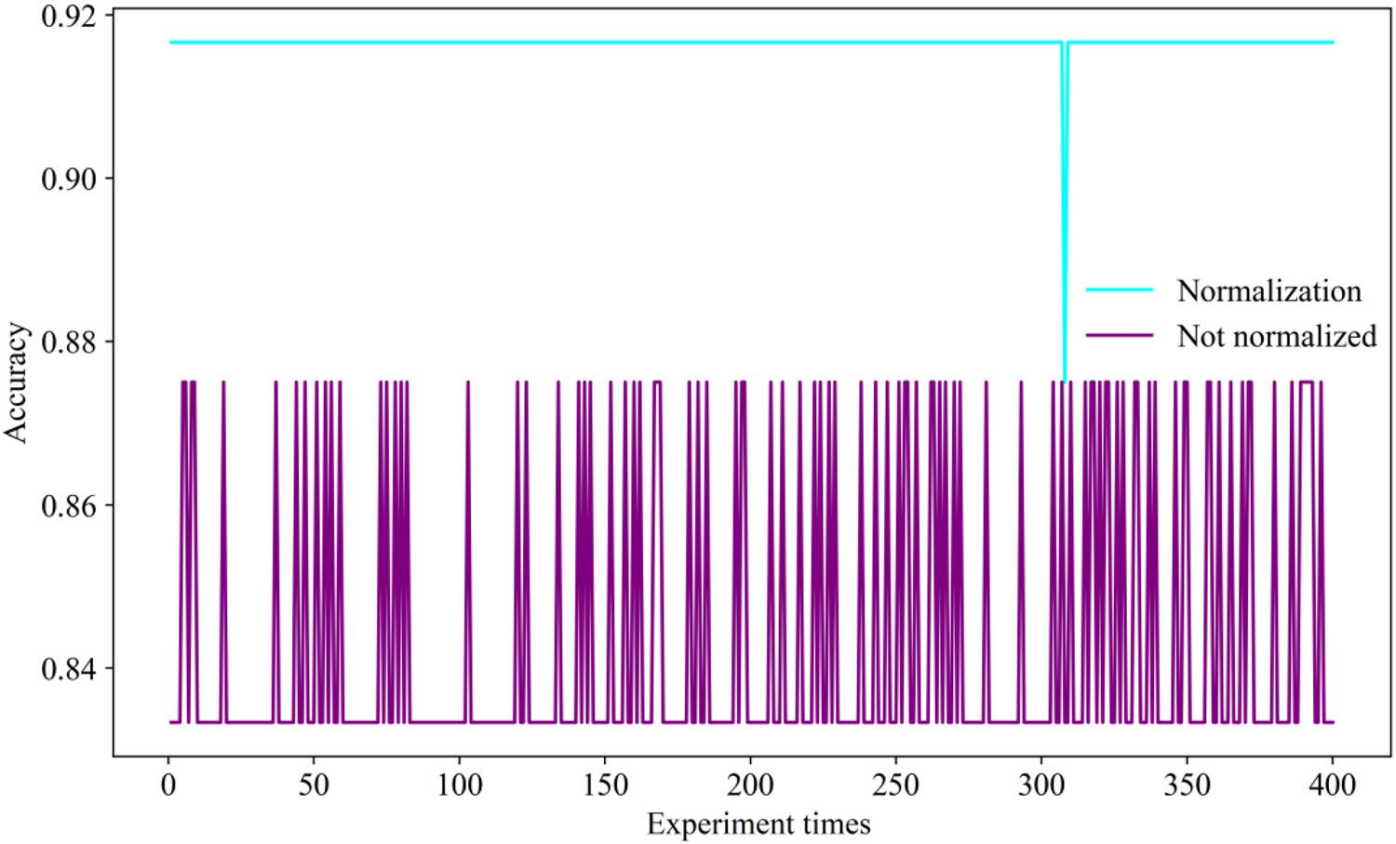
The training accuracy vs experiment times by the data set.

Before the model training, the data of the hydrochemical component should be normalized. Otherwise, it leads to an unstable model training procession. As shown in Fig. 6, the accuracy of the model training is higher after data normalization.

**Figure 7:**
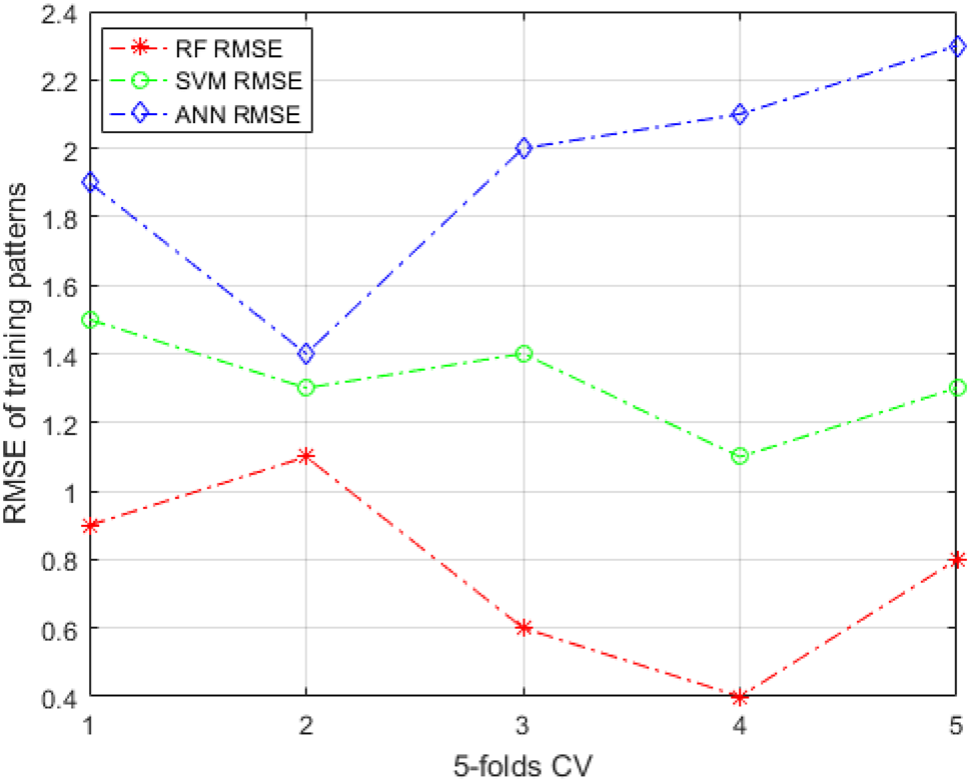
The RMSE curves of training patterns under fivefold CV.

**Figure 8:**
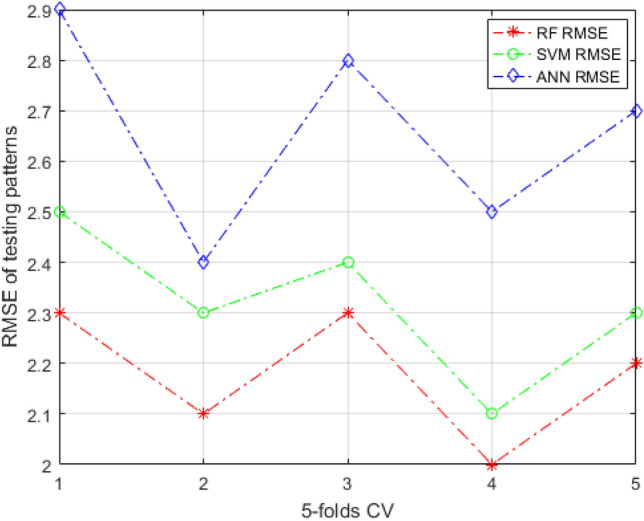
The RMSE curves of testing patterns under fivefold CV.

Figures 7 and 8 illustrate the RMSE curves of the training and testing data set of the RF models under fivefold CV. The RMSE value close to 0 indicates that the error in prediction is marginal. It can be observed that the RMSE of the RF model is smaller than the other two ensemble learning methods, which shows that the prediction performance of RF-based models is better than ANN and SVM in training data set. The curves are more fluctuant in Fig. 8. In addition, all models performed the same trend, which indicates that models are obviously influenced by the data set’s quality. By calculating average values of the RMSE for these models, the RF-based model’s RMSE average value is lower than that of comparison models, implying that the RF-based model has much better performance. Meantime, the k-fold cross-validation is also used for a better evaluation. It splits the training data set into k subsets of equal size, which are named folds. Every fold is used as a validation data set to test the model trees, whereas the right k-1 data set is used for model training. To balance the evaluation result and the training time, in the research, the four-folds is selected.

## Results and discussion

In the random forest algorithm, hyper-parameters optimization has a great influence on the model’s robustness, generalization capability and performance. The step is 1 for max_depth changing from 1 to 100 as well as n_estimator changing from 1 to 100. This sub-section details the selection of optimal hyper-parameters of random forest algorithm.

**Figure 9:**
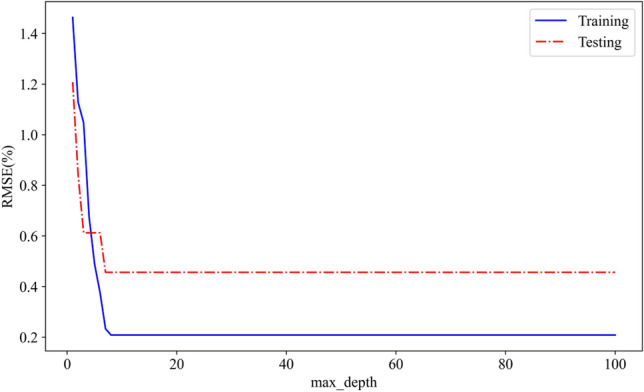
The RMSE curve for the increasing of max_depth.

The max_depth, the maximum depth of each tree, is one of the most important hyper-parameters, which stands for the depth of the tree number. The best number of max_depth has been tested for the model, as is shown in Fig. 9. The blue curve represents the trend for the increasing of max_depth with the training dataset, and the red curve represents the trend for the increasing of max_depth with the validation dataset. From the Fig. 9, we can see that the RMSE value can be the least and keep when the depth of the tree number is 7.

**Figure 10:**
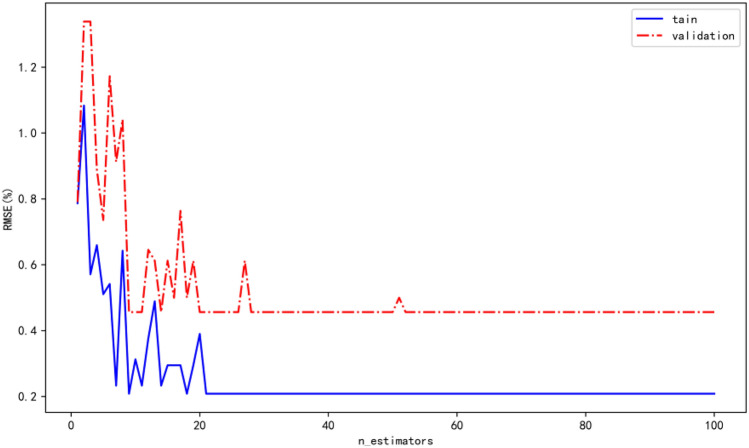
The RMSE curve for the increasing of n_estimator.

To decrease the possibility of over-fitting, the n_estimator is also discussed as another hyper-parameter, which is directly related to the computational cost. A stepwise searching method is used to find optimal values of the model’s n_estimator, as is shown in Fig. 10. From the Fig. 10, we can see that the RMSE value can decrease to 0.2 and 0.5 and remain unchangeable when the n_estimator is 22.

**Figure 11:**
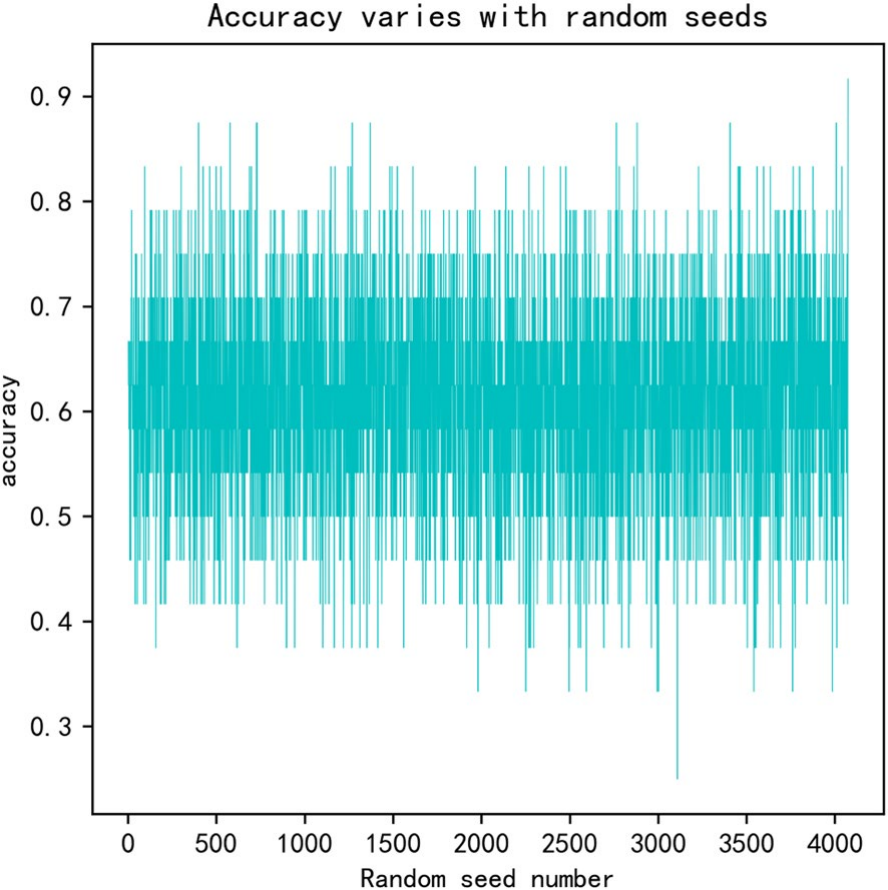
The change of accuracy with different random seed number.

The number of random seeds is another hyper-parameter. The change of accuracy with different random seeds has been tested, as is shown in Fig. 11. We can see that the accuracy can get the highest value when the random seeds number equals 4000.

**Figure 12:**
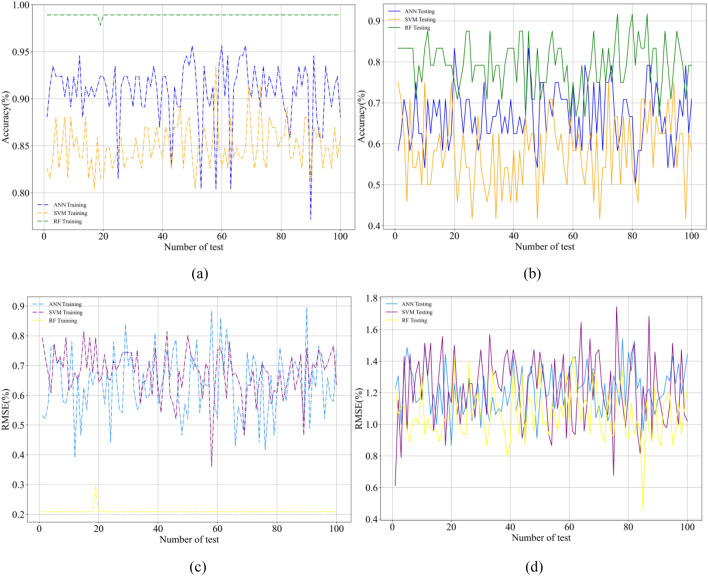
Accuracy and RMSE plot of RF, ANN model and SVM.

**Figure 13:**
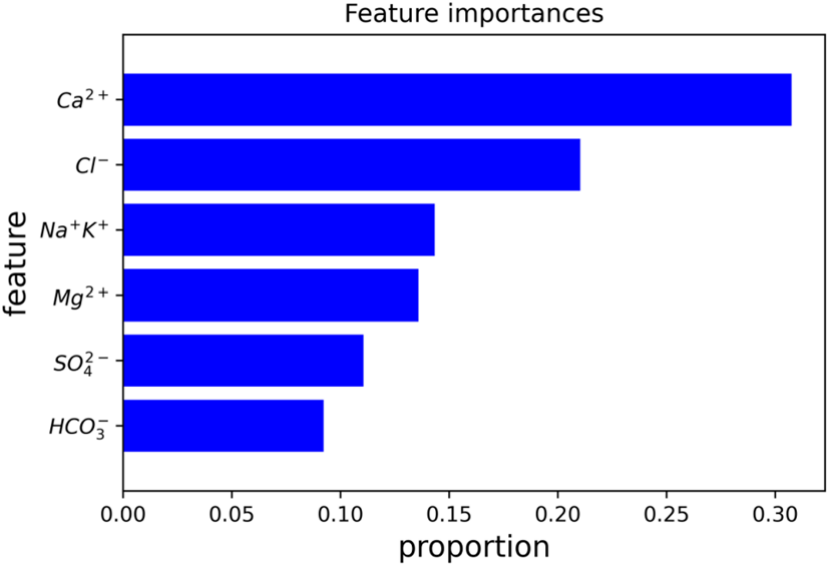
Means (over 10 permutations) of permutation-based variable-importance measures for the explanatory variables included in the random forest model.

The performance of other models is also studied in the manuscript, which is shown in Figs. 12 and 13. The parameters of models are listed in Tables 1 and 2. Figure 12 demonstrates the accuracy (a, b) and the RMSE (c, d) of training and testing of the models (RF, ANN, SVM). According to Fig. 12, one hundred times’ tests have been taken, and the minimum value (0.2 for the training and 0.45 for the testing) of RMSE is calculated by the RF model. The ANN model and SVM model show almost the same performance based on the magnitudes of RMSE. Compared to the other two models, the RF model has the highest accuracy (or best performance) as it exhibits the lowest RMSE and the highest accuracy. In general, the RF model leads to a better match compared to the other two models for the training and testing phases.

**Table 1:** The hyperparameters of the intelligent evaluation of the ANN model.

Number	Parameter	Value
1	Type of model	Sequential model
2	The number of neurons in the input layer	6
3	The number of hidden layer and neurons	2,5
4	The number of neurons in the output layer	5
5	Activation function of hidden layer	ReLU
7	Activation function of output layer	Softmax
8	Epoch	100
9	Learning rate	0.01
10	Optimizer function	Adam
11	Batch_size	10
12	Dropout rate	0.5
13	Error limitation	1*10^–4^
14	Momentum coefficient	0.8

**Table 2:** The hyperparameters of the intelligent evaluation of the SVM model.

Number	Parameter	Value
1	Kernel function	Radial basis function (RBF)
2	Regularization parameter	50
3	Gamma	1/6
4	Cache_size	200
5	Class_weight	1
7	Tolerance	0.001

Feature importance refers to a class of techniques for assigning scores to input features to a predictive model that indicates the relative importance of each feature when making a prediction. Feature importance scores can highlight which features may be most relevant to the target. The trained random forest model can calculate feature importance automatically, which is obtained through the interface feature importance criterion. The gain is calculated by taking each feature’s contribution for each tree, indicating the relative contribution of each feature to the model. Figure 13 shows the six feature variables’ average of feature relative importance (%) under fivefold CV. The blue bars represent the features importance of the RF model. In the current model, Ca^2+^ (33%) is the most important feature variables, followed by Cl^−^ (22%), Na^+^ + K^+^ (15%), Mg^2+^ (14%), SO_4_^2−^ (12%) and CO_3_^2−^ (9%). The result implies significant guidance for exploring the characteristics of mine water.

**Figure 14:**
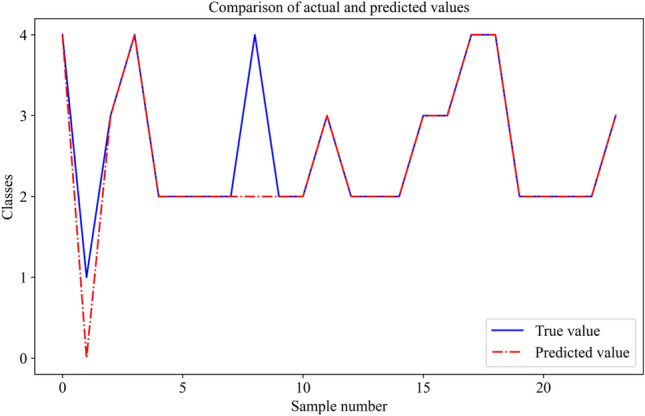
Prediction performance of random forests model.

Twenty-three samples are also used for model prediction. The result is shown in Fig. 14. The blue curve represents the true value of the water source, and the red curve represents the predicting value. There are two error predictions in the twenty-three water samples. It also means that the accuracy of the prediction is 87% by the Random Forests model.

## Conclusions and outlooks

Random forests have developed well over the past years, and are widely accepted as one machine learning approach for a wide variety of tasks. In this study, using mine water data, the random forests model is implemented to develop data-driven predictive models for the source of mine water. Based on the study outcomes, the concluding remarks are listed below:

A hydrochemical dataset was constructed by water sampling from the Pingdingshan coal field, which is divided into five sub-sets for model training and testing. The Random Forests model was trained by 5-folds CV. Compared to SVM and ANN model, the random forests model shows good performances in predicting the source of mine water. 4-folds is the best practice for model training. With the 4-folds CV, a series of hyper-parameter have been tested for the random forests model. For the prediction, the accuracy is 87% by the Random Forests model.

The relative feature importance of source discrimination of mine water can be automatically calculated by the studied random forest model. The VIM indicates that Ca^2+^ of mine water plays the most important role in source discrimination of mine water.

It is also recommended that the random forests model is included as dataset attributes in the predictive models for estimating mine water source. The feature ranking strategy with the machine learning technique might be proper to predict other geological properties for saving geophysical exploration costs. It appears that the study strategies and feature ranking approaches can also be useful to geologists.

## Data Availability

The data that support the findings of this study is available from the Institute of Water Science but restrictions apply to the availability of this data, which was used under license for the current study, and so is not publicly available. Data is however available from the authors upon reasonable request and with permission of the Institute of Water Science.
